# Canadian adaptive platform trial of treatments for COVID in community settings (CanTreatCOVID): recruitment strategies of a decentralized, national randomized controlled trial for acute SARS-CoV-2

**DOI:** 10.3389/fpubh.2026.1698604

**Published:** 2026-02-11

**Authors:** Geil Han Astorga, Andrew D. Pinto, Kawsika Sivayoganathan, Kevin Maruthananth, Banafshe Hosseini

**Affiliations:** 1 Upstream Lab, Li Ka Shing Knowledge Institute, St. Michael’s Hospital, Unity Health Toronto, Toronto, ON, Canada; 2 Department of Family and Community Medicine, St. Michael’s Hospital, Unity Health Toronto, Toronto, ON, Canada; 3 Department of Family and Community Medicine, Faculty of Medicine, University of Toronto, Toronto, ON, Canada; 4 Division of Clinical Public Health, Dalla Lana School of Public Health, University of Toronto, Toronto, ON, Canada; 5 Institute of Health Policy, Management, and Evaluation, Dalla Lana School of Public Health, University of Toronto, Toronto, ON, Canada; 6 Institute of Medical Science, Faculty of Medicine, University of Toronto, Toronto, ON, Canada

**Keywords:** clinical trial, decentralized trial, digital communication, participant recruitment, trial enrollment

## Abstract

**Background:**

Recruitment remains a challenge in clinical trials. This study describes the use of digital and traditional recruitment channels in a national, community-based adaptive platform trial for COVID-19 in Canada.

**Methods:**

Self-reported recruitment sources were collected from participants of a remote, national adaptive platform trial of COVID-19 treatments conducted across six Canadian provinces using a secure web-based application. Recruitment channels were analyzed using descriptive statistics, chi-square tests, and logistic regression to explore associations with province.

**Results:**

From January 2023 to September 2024, 1,515 participants completed the pre-screening process, and 720 were randomized. Of them, 416 were recruited through traditional channels, 303 through digital channels, and one through an undetermined source. Recruitment channels varied by province (*χ*^2^(5) = 81.30, *p* < 0.001; Cramér’s *V* = 0.34), and digital channels were associated with higher odds of recruitment compared with traditional channels (adjusted OR = 2.78, 95% CI 2.20–3.52). The most frequently reported referral source was ‘family, friends, or colleagues’ (20%), followed by provincial government websites and health lines (16.25%), online channels (10.56%), and public communications (9.86%).

**Conclusion:**

Combining traditional and digital recruitment methods supports recruitment. Trials can raise awareness of the study by leveraging both digital and traditional channels and can move information to action through trusted social groups and institutions. Further research is needed to evaluate the cost-effectiveness, reach, and demographic differences across platforms, informing recruitment strategies in future decentralized and adaptive trials beyond COVID-19.

**Clinical trial registration:**

https://clinicaltrials.gov/study/NCT05614349, Identifier NCT05614349.

## Introduction

The COVID-19 pandemic demonstrated the potential of adaptive platform trials (APTs) for rapid evaluation and testing of treatments (1, 2). However, similar to clinical trials (3), platform trial discontinuation due to poor recruitment (4) remains a challenge. Despite the growing availability of trial participation data (5), there is a paucity of consistent reporting on recruitment strategies (6) and outcomes across decentralized clinical trials (7). The lack of sufficient, evidence-based information presents difficulty in understanding effective approaches (8), specifically for the time-sensitive and resource-intensive nature of trials in response to public health emergencies (9).

Previous studies have explored strategies for participant recruitment in primary care settings (10) and of primary care practices (6), yet inconsistent reporting and sparsity of high-quality evidence on effectiveness (3) pose significant challenges for replicating these strategies. Some studies within trials have compared communication platforms and message framing to recruit participants (11, 12), but these efforts have primarily focused on non-communicable diseases such as diabetes and cancer, where the symptomology and trial participation dynamics differ significantly from those of infectious diseases such as SARS-CoV-2. While digital recruitment methods have been applied (13) and mapped (14), there remains a scarcity of data on the strengths and limitations of recruitment strategies, particularly in decentralized clinical trials (7). Additionally, the COVID-19 pandemic accelerated the adoption of digital technologies in healthcare (15), underscoring the potential to understand their value for effective participant engagement.

This study provides a comprehensive overview of the recruitment strategies implemented by the Canadian Adaptive Platform Trial of Treatments for COVID in Community Settings (CanTreatCOVID) and presents the results of traditional and digital recruitment methods. While prior analyses and reviews have examined recruitment outcomes across digital and traditional channels (16–18), this is the first national APT in Canada to report these strategies using participant-reported recruitment data. By describing the strategies applied across six provinces in Canada and their results, this study aims to offer evidence-based insights for recruiting participants and to support the efficient establishment of trials, particularly in public health emergencies.

## Methods

### Study setting

CanTreatCOVID, a national APT, aimed to evaluate the effectiveness of SARS-CoV-2 treatments in reducing symptoms, decreasing emergency department visits and hospitalizations, and preventing post-acute sequelae in non-hospitalized participants. The study operated remotely with a central team based at Upstream Lab, St. Michael’s Hospital in Toronto, Ontario, Canada and planned to enroll participants across Ontario (ON), British Columbia (BC), Newfoundland and Labrador (NL), Quebec (QC), Manitoba (MB), and Alberta (AB). CanTreatCOVID is approved by Health Canada and institutional Research Ethics Boards (REB) of the provinces where the trial operates (https://ClinicalTrials.gov: NCT05614349). The study setting, eligibility criteria, screening process and enrollment of participants are outlined in the protocol (19).

### Patient partner engagement

CanTreatCOVID launched in January 2023 and collaborated with up to 25 patient partners who joined on a rolling basis. They comprised individuals with lived experience of COVID-19 infection and long COVID, healthcare providers, community and organization leaders, and families of individuals with long COVID.

The study team recruited patient partners starting in September 2022 through existing contacts of the Upstream Lab team. In October 2022, a Communications Specialist (GHA) led a 2-h orientation session to discuss the trial’s aims, operations, and eligibility criteria, as well as their role as patient partners. The second hour of the orientation session sought feedback on the consent forms, participant enrollment journey and recruitment channels. Interested patient partners contacted the general CanTreatCOVID email, scheduled an orientation meeting, and received access to a secured online folder with relevant trial documents. The rolling engagement process continued until a Community Engagement Specialist led patient partner onboarding.

Patient partners attended monthly team meetings, in addition to the Recruitment and Communications Committee meetings with research team members. They provided feedback on study materials, recruitment strategies, and community engagement through virtual breakout sessions. Partners received regular recruitment updates via meetings or email. Their feedback and comments were documented, per the Terms of Reference, and reviewed in meetings to track actions taken. Suggestions from patient partners informed the development of the broader recruitment strategy and the identification of specific recruitment channels, including bus ads, radio ads, and community organizations. They were compensated 40 CAD per hour and could withdraw at any time.

### Data collection and evaluation of recruitment strategies

Participant-reported recruitment sources were collected through REDCap during screening. Participants who completed the online pre-screening form were screened by research assistants over the phone and were asked to identify one pre-determined recruitment channel from the following options: “a letter mailed to my residence,” “CanTreatCOVID Online Self-Screening form,” “CanTreatCOVID website,” “Community pharmacy,” “Dicentra/Vitalabs clinic,” “My primary care provider,” or “Other.” Participants selecting “Other” were asked to provide additional details in a comment field to identify the source of recruitment.

Recruitment data were exported from REDCap and organized by province and recruitment channel. Responses from participants who selected “Other” as their recruitment source were analyzed by GHA using qualitative content analysis (20). Free-text responses for “Other” were reviewed to identify recurring concepts and determine alignment with predefined recruitment categories. For responses that did not correspond to existing options, new categories were developed, including: Family, friends or colleagues; Provincial health lines; Rapid Antigen Test (RAT) kits; Radio; Hospital/healthcare centres; Nurses/nurse practitioners; Community centre; Another study; Bus ads; Church ads; Conferences; Cold calls; Provincial government websites; Search engine ads; Advertising websites; Online forums; Email; Email—COVaxON. Responses indicating “email” and “[hospital/clinic name] email” were combined, while a separate category was created for those in ON who explicitly linked their email source to consent given at the time of vaccination, classifying them under the COVaxON email list. Participants recruited through the CanTreatCOVID Emergency Department Sites were also recorded. Ambiguous responses, such as references to unspecified websites, were grouped into broader categories of “Website” or “Online” to reflect the exact text response of the participants, and one item was classified as “Undetermined” for a participant who could not recall the recruitment source. All responses were subsequently re-coded.

Descriptive statistics were used to summarize screened individuals, recruited participants and conversion rates per channel. Chi-square tests analyzed the distribution of digital and traditional channels for recruitment across provinces, with effect size quantified using Cramér’s V. An exploratory logistic regression model was fitted with recruitment status as the dependent variable to estimate adjusted odds ratios (aORs) with 95% confidence intervals, examining associations with recruitment channel while adjusting for province. Participants with an undetermined recruitment source were excluded from analyses involving recruitment channels. Statistical analyses were conducted using IBM SPSS Statistics version 31.0.1.0, with significance defined at *p* < 0.05.

Digital channels are defined as recruitment sources that use electronic and online media (14), including online platforms and forums; websites such as the provincial governments’ official pages, CanTreatCOVID website, and advertising websites; social media; emails; search engine advertisements; the study’s online pre-screening form; radio ads; cold calls; and online news media outlets. In contrast, traditional channels refer to sources that rely on in-person communication methods or physical materials, including family, friends or colleagues’ referrals; public communications such as posters or flyers; emergency departments, health centres, community centres and clinics that partnered with a contract research organization (CRO); provincial health lines; pharmacists or nurses’ referrals; mailed letters; RAT kits; bus or church ads; and conferences. The classification of cold calls under digital channels is based on the use of an electronic system to identify phone numbers, whereas calls to provincial health lines are categorized under traditional channels due to the lack of automation involved, consistent with the approach used in a previous study (14).

### Recruitment methods

This section outlines the strategies implemented by CanTreatCOVID to reach potential participants in the trial, including those that did not yield recruitment. The ideas were generated in collaboration with the patient partners, research team members, CanTreatCOVID staff and committee members. All strategies were approved by the respective REBs in each province with active CanTreatCOVID sites.

#### Mailed letters

Research coordinators at each Practice-Based Research Network (PBRN) affiliated with CanTreatCOVID received REB approval to acquire a list of potentially eligible participants for the study using electronic medical records. The study team prepared pre-signed, undated letters to mail to the residences of participants from December 2023 to August 2024. Potential participants were sent a one-time invitation letter that contained information about the study and its eligibility criteria.

#### Online pre-screening form

All CanTreatCOVID provincial sites, except QC, received REB approval to use a secure pre-screening form built on REDCap. Participants chose their preferred language (English or French), province of residence, whether they have experienced symptoms within the past 5 days, whether they have used an antigen or polymerase chain reaction (PCR) test, their age, any chronic conditions they may have if aged 49 or younger, and their preferred contact method, either by phone or email. The pre-screening form link was on the CanTreatCOVID website and printed on materials as a QR code.

#### Community pharmacy

From January 2023, a one-time fax campaign distributed a customized one-page information sheet to over 5,000 pharmacies across participating provinces, using regulatory contact lists. The sheet highlighted benefits for pharmacists, including reduced screening burden, advancing treatment options, and providing personalized care to participants. Research pharmacists and assistants conducted follow-up outreach via emails, phone calls, and in-person visits to select pharmacies in highly populated areas to distribute flyers and rapid antigen test kits labelled with QR-coded study information. Pharmacists were reimbursed 40 CAD for each eligible participant they referred.

#### Contract research organization

In October 2023, CanTreatCOVID engaged a CRO to support participant recruitment efforts. A request for proposal was distributed via social media and email, resulting in two submissions. Following Unity Health Toronto’s policies, the selection process included meetings with both companies and a scoring assessment conducted by a Co-Principal Investigator, a Senior Research Coordinator, and a Communications Specialist. After selecting the CRO, the study team held bi-weekly meetings for recruitment updates. The CRO employed various strategies, including email outreach, in-person clinic recruitment, and social media and paid media advertising.

#### Partnership with primary care providers

Partnerships with primary care providers (PCPs) and PBRNs varied across provinces. In AB, no formal partnerships were established, as efforts to engage clinics were unsuccessful; however, some PCPs were informed about the study and invited to display posters. No partnerships with PCPs or PBRNs were formed in BC and NL. ON engaged over 200 clinics through both passive and proactive recruitment strategies. These efforts included outreach via email, postal mail, posters, newsletters and presentations at clinic rounds, with engagement spanning from February 2023 to January 2025.

#### Provincial websites and health lines

CanTreatCOVID obtained permission to feature the study on provincial government websites. Approved postings included a brief study description, website link, and contact details, placed within COVID-19 treatment-related webpages or displayed as notifications. Approval and implementation varied across provinces, with active website postings maintained from June 2023 to September 2024. In some provinces, individuals who called government health phone lines for COVID-19 support were informed about and referred to the study.

#### Digital media advertising

Digital ads included setting up search ads on Google using REB-approved materials to determine English and French keywords and phrases. The ads were linked to the CanTreatCOVID website or pre-screening form and ran from November 2023 to September 2024. The budget fluctuated, with the highest allocation during peak times of high COVID-19 rates in Canada.

Social media ads were active on Facebook, Instagram, and LinkedIn from January 2023 to September 2024, featuring study posters, infographics on flu and COVID-19 vaccinations, videos from patient partners and researchers, and animated lay-language study materials. All posts included the study website, pre-screening form link or contact information. For X, the study team contacted health professionals with more than 10,000 followers to post about CanTreatCOVID on their timeline or retweet it from the Upstream Lab account. The locations of active ads were updated to coincide with the launch of CanTreatCOVID in each province. A YouTube account was created to showcase the individual videos and montage created by the study team and patient partners, and to run awareness ad campaigns.

The study was posted on a Canadian online classified advertising website and a healthcare professional’s website. Partner institutions, including community organizations, research centers, and universities, added the study information to their websites.

#### Email

The Ontario Ministry of Health provided a list of over one million contacts of individuals who had consented to be contacted about COVID-19-related research when receiving their COVID-19 vaccinations. Obtaining access, while ensuring compliance with REB and institutional approvals, took over 6 months to complete. Once granted, the list was uploaded to Unity Health Toronto’s secure email platform. Contacts were segmented to optimize email delivery, protect the sender’s IP reputation, and minimize the risk of spam. After each campaign, the list was updated to maintain compliance with privacy policies and contact preferences. The email included a reason for contact, detailed study information, steps to participate and its benefits, contact information, the pre-screening form link, an unsubscribe button, and the CanTreatCOVID mailing address. Research staff and investigators in other provinces also shared the study through their professional networks via email.

#### Media engagement

CanTreatCOVID PI (AP) and a Co-PI (BH) provided expert comments on COVID-19-related news stories mentioning the study. AP and GHA participated in interviews conducted by a multicultural news channel in Canada to reach diverse communities. A patient partner who is an emergency department physician wrote a story about long COVID and mentioned CanTreatCOVID. The study team also ran radio ads in NL, ON, and QC and participated in podcast interviews by well-known health broadcasters to reach potential participants.

#### Traditional media advertising

Advertisements were placed in liturgical publications across five urban cities in ON and ran for 1 year. Each advertisement included a brief description of the study, eligibility criteria, and contact information. The study information was also promoted through a media outlet in QC, with advertisements printed in local newspapers and published on the media outlet’s website.

The study was promoted through bus ads in ON, NL, AB and BC and train ads in ON.

#### Community engagement

Community Engagement Specialists in ON, BC, and AB led the initiatives in their provinces and informed the national strategy. They cultivated partnerships with community organizations, attended events to promote the study, distributed posters and RAT kits, and facilitated collaboration with patient partners. The specialists worked with libraries, centres, shelters and retirement associations to reach underserved populations and increase awareness of the study.

## Results

From January 2023 to September 2024, a total of 1,515 participants completed the pre-screening process. Among them, 720 participants were randomized to either the usual care or the Nirmatrelvir/ritonavir arm of the study (AB: 86, BC: 212, MB: 10, NL: 53, ON: 348, and QC: 11). Table 1 outlines the number of participants screened and recruited through each channel, the percentage that each channel contributed to the total recruitment, and the conversion rate for each channel, showing a higher number of participants recruited through traditional channels (57.78%) compared to digital channels (42.08%), with the remaining percentage attributed to one participant who was recruited but could not recall the source.

**Table 1:** Recruitment results per channel.

Traditional channels	Screened	Recruited	% of total	Conv. rate %	Digital channels	Screened	Recruited	% of total	Conv. rate %
Family, friends, or colleagues	245	144	20	58.78	“Online”	95	76	10.56	80
Public communications	174	71	9.86	40.80	Provincial government websites	83	58	8.06	69.88
Provincial health lines	140	59	8.19	42.14	Social media	104	44	6.11	42.31
Participants’ primary care provider	86	42	5.83	48.84	Email: COVaxON	87	35	4.86	40.23
CanTreatCOVID emergency department site	25	24	3.33	96	Email	74	25	3.47	33.78
Contract research organization	39	22	3.06	56.41	Search engine ads	75	20	2.78	26.67
Community pharmacy/pharmacist	45	18	2.50	40	CanTreatCOVID website	77	26	3.61	33.77
Letter	57	18	2.50	31.58	Pre-screening form	36	12	1.67	33.33
RAT kits	9	6	0.83	66.67	Radio ads	4	2	0.28	50
Hospital/healthcare Centres	8	3	0.42	37.50	“Website”	3	2	0.28	66.67
Nurse/nurse practitioners	8	4	0.56	50	Advertising website	3	1	0.14	33.33
Community centre	1	1	0.14	100	Cold calls	3	1	0.14	33.33
Another study	2	1	0.14	50	Online forum	2	1	0.14	50
Bus ads	4	1	0.14	25	News media	1	0	0	0
Church ads	1	1	0.14	100					
Conferences	1	1	0.14	100					
Sub-total	845	416	57.78	49.23	Sub-total	647	303	42.08	46.83
Undetermined channel	Screened: 23			Recruited: 1		Conv. rate: 4.35%			
Total	Screened: 1515			Recruited: 720		Conv. rate: 47.52%			

### Overview of recruitment channels per province

Recruitment sources varied by province. Chi-square tests indicated moderate association between province and recruitment channel, *χ*^2^(5, *N* = 719) = 81.30, *p* < 0.001, Cramér’s *V* = 0.34 (Table 2). In AB, most of the participants (48) were recruited through the provincial health line that residents could call for COVID-19-related inquiries. BC participants primarily learned about the study “online” (73) without providing further details and through family, friends, or colleagues (45). Most of the MB participants found out about the study through their primary care provider (4). The sources of recruitment in NL are primarily referrals from family, friends or colleagues (21) and public communications such as posters or advertisements (19), similar to the data in ON that showed family, friends, or colleagues as the most common source (62), followed by public communications (45). More than half of the 11 participants in QC indicated family, friends and colleagues (6) as their source.

**Table 2:** Distribution of recruitment channels per province.

Province	Traditional, *n* (%)	Digital, *n* (%)	Total			
ON	194 (55.7)	154 (44.3)	348			
BC	86 (40.6)	126 (59.4)	212			
AB	74 (86.0)	12 (14.0)	86			
NL	49 (92.5)	4 (7.5)	53			
MB	7 (70.0)	3 (30.0)	10			
QC	6 (60.0)	4 (40.0)	10			
Total	416 (57.9)	303 (42.1)	719^a^			

a One participant with an undetermined recruitment source was excluded.

### Raising awareness of the study

While unquantifiable, it is important to note the outreach efforts of the study team to raise awareness of CanTreatCOVID. Community engagement, including attendance at community events and academic conferences, sparked conversations about the study that may have contributed to the increased referrals from family, friends or colleagues in different provinces. From January 2023 (launch of Ontario: first CanTreatCOVID provincial site) to September 2024 (completion of the Nirmatrelvir/ritonavir arm), the CanTreatCOVID website received a total of 87,533 visits. The top 10 website traffic sources were unknown (28,827), Google (12,725), third-party advertising websites and applications affiliated with Google (4,883), Facebook (2,696), Twitter (1,230), VOCM local news (1,235), https://www2.gov.bc.ca (653), newsletter (383), https://calgarycitizen.com (365), and Bing (272).

### Overview of recruitment channels

Recruitment outcomes varied across channels. An exploratory logistic regression adjusting for province showed that recruitment channel was significantly associated with recruitment. Participants who reported digital channels as their source had higher odds of recruitment compared to those who indicated traditional channels (adjusted OR = 2.78, 95% CI 2.20–3.52, *p* < 0.001).

While referrals from family, friends or colleagues led to the highest number of participants enrolled (144), it is unclear how referees discovered the study. Of the referrals, 14 participants were referred by family members who learned about the study through digital means, but were categorized as family, friends or colleagues, since they were the final point of contact considered as the recruitment source. Example text inputs are “wife got an email” and “Family member-Aunt heard about it on [the] radio.” Traditional channels also informed family members who subsequently referred two participants to the study: noticed a poster at a university lab (1) and learned about the study at conferences (1). Other family member referrals were difficult to trace back to either a traditional or digital channel. For instance, a participant who responded “Family member who is a pharmacist” may have learned about the study through faxes sent to pharmacies or in-person outreach by team members. There were also family members who “heard about” the study at a hospital, which may refer to a poster in a waiting room, an email from their primary care provider affiliated with a hospital, or a direct referral from a clinician.

Participants indicated “online” (76) and “website” (2) as their recruitment source without further information. Public communications (71) included discovering the study through posters and advertisements. Engagement with provincial officials yielded recruitment via COVID-19-related webpages (58) and health line (59). Some reported nurses or nurse practitioners (4) as their source. Participants indicated receiving an email or seeing a poster in hospitals or health centres (3).

Digital channels, including social media (44), emails (22) and emails through the database of people who consented to be contacted when receiving vaccines (35) and search engine ads (20), funneled participants. The CanTreatCOVID website (23) and pre-screening form (12) were also sources of recruitment. PCPs of participants (42), emergency departments (24) and community pharmacists or pharmacy (18) referred participants to the study. The remaining methods were through the CRO (21), mailed letters (19), stickers on RAT kits (6), and radio advertisements (2). One participant each was recruited through another study, bus ads, church ads, cold calls, community centres, conferences, advertising website, and online forum, in addition to one undetermined source.

## Discussion

This article provides a detailed account of the various recruitment strategies employed by CanTreatCOVID, a national and decentralized clinical trial of COVID-19 treatments, and aims to support researchers in replicating the findings for future work. Results from six Canadian provinces show higher recruitment yield due to support from government health officials in promoting clinical trials, as well as the role of family, friends, colleagues, and public communications in participant recruitment. Collaboration with patient partners, community engagement, and participation in conferences or other events contributed to raising awareness of the study.

Trust is a crucial factor in clinical trial recruitment. Occa et al. (25) found that trust in social support networks, including family and friends, as well as healthcare providers who incorporate health messages from government sources and health organizations, plays a key role in shaping trust and understanding of clinical trials. In CanTreatCOVID, family, friends, and colleagues were the primary sources of recruitment, highlighting the importance of trusted relationships between message sources and recipients in trial communication. Family members may have learned about the study through digital channels, since the 87,533 visits to the CanTreatCOVID website originated from various online sources, and that the trust among referees (family members, friends, and colleagues) may have helped move participants from awareness to action, i.e., study participation. Similar to studies in a systematic map that added digital tools for recruitment to address inadequate enrollment through traditional methods (14), and a decentralized trial that reported success with a combined digital and traditional recruitment strategy (7), there is a possibility to integrate both digital and traditional channels to enhance recruitment. In CanTreatCOVID, digital channels facilitated mass outreach to potential participants, suggesting that these can be combined with traditional channels—such as referrals from family or friends—to further support recruitment.

Other recruitment strategies that yielded high results were provincial health lines and provincial government websites, underscoring the pivotal role of credible sources in transforming awareness into action (21, 26). Public communications, as one of the most common recruitment source, included printed materials distributed through trusted community health centers, clinics, libraries, and support organizations, as well as by community leaders and organizations.

The increased utilization of online platforms during the COVID-19 pandemic introduced affordances for mixed traditional and digital recruitment in trials (13, 24). While leveraging various channels reaches broad audiences, understanding geographical and other relevant contexts is equally important for successful recruitment (22, 23). The observed variation in traditional and digital recruitment channel distribution across CanTreatCOVID sites aligns with the moderate association identified in the analysis. Two of the provinces that generated 77% of the recruitment results had different primary sources: traditional for ON and digital for BC, whereas other sites relied on traditional channels. The heterogeneity across provinces suggests that geographical and other characteristics influence recruitment rather than uniform reliance on a single recruitment strategy, and highlights the potential complementary role of traditional and digital channels. A key limitation of this analysis is that recruitment strategies were not randomized, and participants may have been exposed to multiple recruitment channels. As a result, the findings should be interpreted as descriptive comparisons and associations rather than causal or inferential effects of specific recruitment approaches.

This study reports on a wide range of recruitment channels and provides insights into their effectiveness in engaging with potential participants. Future decentralized trials may refer to these findings to determine key strategies for utilizing traditional and digital recruitment methods, particularly during public health emergencies where rapid recruitment and assessment of treatments are crucial. The self-reported fields on recruitment that led to ambiguity in some responses are also a limitation. In BC and ON, participants indicated “online” or “website” on the pre-screening form without specifying whether they were referred through an internet search engine, the CanTreatCOVID website, a provincial government site or other online sources. In ON, some participants noted “email” without clarifying whether it came from their primary care provider or the CoVaxON email database. The focus on COVID-19 outpatient treatment trials and the public discourse surrounding COVID-19 at the time of the study, which may have influenced participation, could also limit the generalizability of the findings.

The integration of both traditional and digital strategies appears to enhance participant recruitment. Trials can raise awareness of the study by leveraging both types of channels and move information to action through trusted social groups and institutions. Future studies may compare the effectiveness of platforms beyond COVID-19 decentralized trials to provide insights into optimizing traditional and digital recruitment strategies for tailored recruitment of diverse populations.

## Data Availability

The raw data supporting the conclusions of this article will be made available by the authors upon reasonable request.
